# Mining functional subgraphs from cancer protein-protein interaction networks

**DOI:** 10.1186/1752-0509-6-S3-S2

**Published:** 2012-12-17

**Authors:** Ru Shen, Nalin CW Goonesekere, Chittibabu Guda

**Affiliations:** 1 Department of Genetics, Cell Biology and Anatomy, University of Nebraska Medical Center, Omaha, NE, 68198, USA; 2 Department of Computer Science, State University of New York at Albany, 1400 Washington Ave., Albany, NY 12222, USA; 3 Department of Chemistry and Biochemistry, University of Northern Iowa, Cedar Falls, IA 50614, USA; 4 Bioinformatics and Systems Biology Core Facility, University of Nebraska Medical Center, Omaha, NE, 68198, USA

## Abstract

**Background:**

Protein-protein interaction (PPI) networks carry vital information about proteins' functions. Analysis of PPI networks associated with specific disease systems including cancer helps us in the understanding of the complex biology of diseases. Specifically, identification of similar and frequently occurring patterns (network motifs) across PPI networks will provide useful clues to better understand the biology of the diseases.

**Results:**

In this study, we developed a novel pattern-mining algorithm that detects cancer associated functional subgraphs occurring in multiple cancer PPI networks. We constructed nine cancer PPI networks using differentially expressed genes from the Oncomine dataset. From these networks we discovered frequent patterns that occur in all networks and at different size levels. Patterns are abstracted subgraphs with their nodes replaced by node cluster IDs. By using effective canonical labeling and adopting weighted adjacency matrices, we are able to perform graph isomorphism test in polynomial running time. We use a bottom-up pattern growth approach to search for patterns, which allows us to effectively reduce the search space as pattern sizes grow. Validation of the frequent common patterns using GO semantic similarity showed that the discovered subgraphs scored consistently higher than the randomly generated subgraphs at each size level. We further investigated the cancer relevance of a select set of subgraphs using literature-based evidences.

**Conclusion:**

Frequent common patterns exist in cancer PPI networks, which can be found through effective pattern mining algorithms. We believe that this work would allow us to identify functionally relevant and coherent subgraphs in cancer networks, which can be advanced to experimental validation to further our understanding of the complex biology of cancer.

## Background

Protein-protein interaction (PPI) networks carry vital information on the molecular functions and biological processes of cells. Analysis of PPI networks associated with specific disease systems including cancer helps us to better understand the complex biology of diseases. PPI networks are dynamically modulated in a tissue-specific microenvironment; hence, a set of similarly expressed genes from two types of cancer tumors may exhibit different PPI patterns. A lot of gene expression data has been accumulated on cancer-specific tumors warranting the need for developing effective algorithms to translate the differentially expressed gene lists into functionally coherent modules that are common to all cancers or shared in a given subset of cancers. To achieve this, genes are mapped to corresponding proteins and known PPIs are represented as a network graph for further analysis. Using graph theory-based algorithms, pairs of networks can be compared to identify common, distinct or frequent sub-networks. These sub-networks containing a set of proteins (nodes) with a distinct set of connections (edges) can represent a functional unit in a pathway or in a biological process. Similarly, frequent sub-networks (network motifs) may represent recurring functional units within a network or among multiple networks. In this study, we focus on developing a graph-based algorithm to identify common and frequent network motifs from PPI networks of nine different cancers.

Graphs have been widely used to model a variety of data types such as PPI networks [1], biological pathways [2] and molecular structure of chemical compounds [3]. Graph comparison has a wide range of applications in biological data analysis. For example, by aligning biological pathways represented by graphs, evolutionarily conserved patterns are identified [2]. Similarly, by measuring the discrepancies between PPI networks of healthy and sickened individuals, interactions that are involved in disease outbreak and progression are determined [4].

Existing methods for graph comparison can be categorized into the following three major types: distance-based, alignment-based and kernel-based methods. In a distance-based method, similarity of graphs is measured based on the graphs' common structures [5,6]. The larger a maximum common subgraph (MCS) is, the more similar are the two graphs; and thus the smaller the MCS distance between the graphs is. The MCS distance between the graphs is defined to be 1-|*V_mcs_*|/{|*V*_1_|, |*V*_2_|} where |V| is the number of nodes in graph G = (V, E) [5]. The MCS distance method only considers the maximum common subgraph when comparing graph similarity. It will only identify graphs that globally resemble each other and ignore graphs that share many similar but disconnected subgraphs. Another distance-based method [7] measures the similarity of graphs based on their edit distance. With substitutions, deletions and insertions for both nodes and edges, any graph can be transformed into another graph by iteratively applying these operations. Intuitively the more operations needed, the more dissimilar the two graphs are. With a cost function associated with each operation, the graph edit distance is defined to be the minimum total cost to transform one graph to the other. However, similar to the MCS method, the edit distance methods also measure only the global similarity of the graphs.

The alignment-based methods utilize the idea of graph alignment that is conceptually similar to sequence alignment. In sequence alignment, different scores or penalties are assigned for matches, mismatches and gaps, and the alignment algorithm looks for the best way to arrange the sequences so that the overall alignment score is maximized. In graph alignment, the similarities of graphs are determined by the conservation of interactions, which is measured through the edges and similarity of nodes [8,9]. Depending on the requirement, the node-based or edge-based weights are used in calculating the alignment score [8]. Graph alignment algorithms such as PathBLAST [2] use the dynamic programming approach to find optimum solutions. Graph alignment algorithms can detect global or local similarity depending on the scoring function used by the algorithm. However these algorithms either end up with exponential running time or turn to heuristic methods for solutions when dealing with graphs that contain cycles.

The third approach, using kernel-based methods measures graph similarities through kernel functions. Existing graph kernels can be viewed as a special case of R-convolution kernels proposed by Haussler [10]. The basic idea of a graph kernel is to decompose a graph into smaller substructures, and build the kernel based on similarities between the decomposed substructures. The natural and most general R-convolution on graphs would decompose graphs to all of their subgraphs and compare each pair of the subgraphs. However, it is proven in that computing all-subgraph kernel is as hard as deciding subgraph isomorphism which is NP-hard [11]. Alternative graph kernels include product graph kernel, marginalized kernel, subtree-pattern kernel, and so on. These kernels differ in the way they decompose graphs to substructures and the similarity measure they use to compare the substructures. Similar to distance-based methods, kernel methods can only be used to measure global similarity of graphs. There is no information about which subgraphs contribute to the similarities.

One of the most important tasks in the analysis of PPI networks is to predict functional modules that represent either stable protein complexes or groups of transiently interacting proteins that together can accomplish a biological function. These functional modules can be mapped to specific subgraphs in PPI networks. Below, we discuss three methods that have been used to extract substructures from graphs: (i) frequent subgraph identification, (ii) graph segmentation and (iii) core-based clustering. *Apriori*-based approach and pattern growth approach are the two major types of algorithms for identifying frequent subgraphs. The discovery of frequent subgraphs usually consists of two steps that include candidate generation and frequency counting. *Apriori*-based algorithms such as FSG [12] generate candidates of larger size by joining two smaller subgraphs. In order for two frequent k-subgraphs to be eligible for joining, they must contain the same (k-1)-subgraph. This introduces a lot of overhead, as there are multiple ways to join two subgraphs of size k. The frequency verification step involves subgraph isomorphism test and therefore is not feasible for large graphs. On the other hand, the pattern growth approach [13] extends patterns from a single pattern directly, instead of joining two smaller subgraphs. Pattern growth approach needs to deal with the redundancy problem: the same (k+1)-subgraph can be generated from extending many different k-subgraphs. Both *apriori*-based approach and pattern growth approach are restricted by the graph size due to the subgraph isomorphism problem. Heuristic methods such as Subdue [14] look for incomplete result set. Subdue is an approximate algorithm and finds patterns that can best compress the original graph by substituting those patterns with a single vertex. Minimum description length (MDL) is used to evaluate how efficient the graph can be compressed.

Graph segmentation method extracts substructures by partitioning graphs into disjoint dense subgraphs. K-means clustering [15] aims to partition graphs to clusters that minimize the within-cluster sum of squares. Min-cut [16] and a more recent spectral clustering algorithm [17] consider not only the within-cluster density but also inter-cluster distance. King et al. [18] used a cost-based local search algorithm to find highly interconnected subsets of nodes.

In contrast to the graph segmentation method, where the central nodes of the subgraphs are usually randomly chosen, in core-based clustering the central nodes are selected before clustering is performed [19,20]. The central nodes are also referred to as seeds or core of substructures. MCODE method [1] selects the central nodes based on the highest k-core of the nodes neighborhood. A k-core is a graph of minimal degree k. All nodes are weighted based on their local network density using the highest k-core of the nodes neighborhood. SPICi method proposed by Jiang and Singh [19] chose the nodes that have highest weighted degree as core nodes. After selecting the central nodes, clusters are expanded to maximize the local density of the substructures. The expansion stops when local density stops increasing or when all nodes are exhausted.

Due to the NP-hardness of many graph problems, most of the previous methods offer approximate solutions to measure graph similarity. In this paper we present a method that produces the exact solutions in graph comparison and pattern identification. Our algorithm works in a bottom up fashion. It starts from one-node subgraph, and proceeds to one-edge and multiple-edge subgraph. At each loop the search space is reduced by eliminating parts of networks that are not eligible for next round of comparison. Even though the run-time increases exponentially as the size of subgraph increases, in our case the size of the search space, as the base of the exponential, reduces quickly. Therefore we can obtain the complete result in a reasonable amount of time. As we look for common substructures across the networks, we also perform graph isomorphism test. Graph isomorphism problem is known to be in NP; however, it's unknown to be in P or NP-complete if P ≠ NP. In our specific context of network comparison, we solve this in polynomial time with our pattern-labeling algorithm.

We applied our algorithm on nine cancer associated PPI networks to identify common and frequent patterns in these networks. We collected differentially expressed genes from microarray studies of various solid tumor tissues derived from the Oncomine database [21]. Using the algorithm we identified common frequent subgraphs of up to 10 edges in these networks. These subgraphs may correspond to functional modules that play common roles in cancer diseases as they occur multiple times in all the nine cancer networks.

## Results and discussion

### Cancer protein interaction networks

Our PPI networks are constructed from a comprehensive, non-redundant dataset of experimentally-derived PPIs [22] that are collected from five major databases including IntAct [23], MINT [24], HPRD [25], DIP [26] and BIND [27]. Our goal is to mine for cancer-associated subgraphs from the global interaction networks; however, PPI data that are specific to a cancer tumor do not exist in the public domain. Hence, we used all the available PPI datasets for humans from five major databases as the basis for our studies. In our final human PPI network, there are 19,710 unique proteins representing 95,931 unique interactions. Note that this unique set of proteins exhibit some level of redundancy because splice variants with minimal sequence differences are included as unique proteins due to the fact that PPIs are isoform-specific.

We collected differentially expressed genes (DEGs) between tumor and normal samples from microarray studies of nine different solid-tumor cancer types using the Oncomine database [21]. Oncomine is a cancer microarray database that provides access to DEGs on most major types of cancer. For each cancer, DEG lists are available from multiple experiments, where the q-values (a variant of p-value) for a gene vary from experiment to experiment. Hence, we choose only DEGs whose average q-values are equal to or smaller than 0.05. The gene lists are then mapped to protein lists using our in-house mapping tools. The number of proteins is roughly 2 times the number of genes due to the multiple mappings between genes and proteins. These proteins are further mapped to the proteins in the human PPI network to create nine cancer-specific PPI networks. Table 1 summarizes the number of genes and proteins and the corresponding network size associated with each cancer type.

**Table 1:** Number of genes and proteins mapped under each cancer network.

Cancer type	Number of genes	Number of proteins	Edge count	Node count
Bladder cancer	11771	29286	47909	10726
Breast cancer	11373	26498	33558	8611
Cervical cancer	9811	22447	19332	6288
Colorectal cancer	18982	40905	58212	13273
Esophagus cancer	5135	13380	13405	4218
Gastric cancer	12137	28224	41289	9707
Melanoma	8763	22421	30843	7677
Pancreatic cancer	17339	37160	52125	12199
Prostate cancer	11181	27598	41658	9621

**Figure 1:**
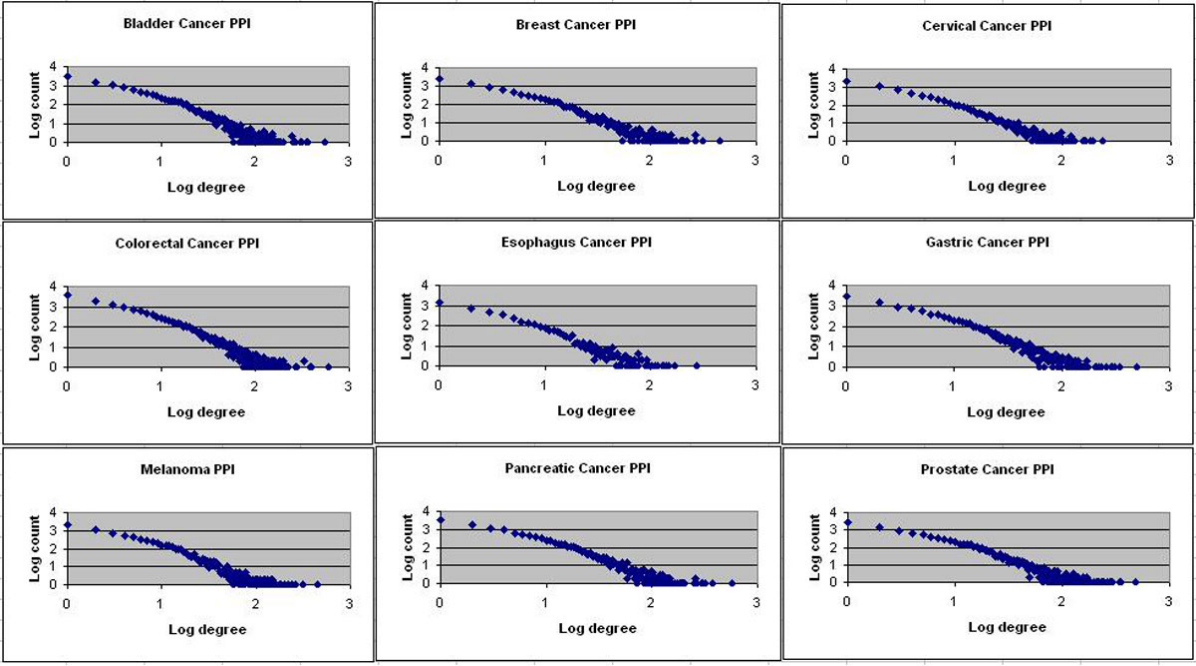
**Power-law distribution of PPI networks from nine different cancers**.

Similar to many PPI networks, cancer PPI networks also exhibit power-law degree distributions (Figure 1). Such a distribution indicates that most proteins in the network have only a few interactions, while a small number of proteins acting as hubs participate in a large number of interactions. This makes cancer PPI networks resistant to random failure but vulnerable to targeted attacks to the hub nodes. Figure 1 depicts the degree distribution (on a log-log scale) of the nine cancer PPI networks we studied. All of the charts exhibit a linear relationship on a log-log scale, which is the signature of power-law distribution.

### Network analysis

The reason we are interested in frequent patterns is that the presence of these subgraphs in PPI networks constitute an analogy to motifs in multiple sequence alignment. These frequent subgraphs represent conserved functional modules that play significant roles in the disease systems we study. First we look for frequent subgraphs within a network because of the possibility of finding more than one identical subgraph from nodes that belong to the same cluster (see below). Then we perform comparative analysis across multiple networks to measure the commonality across networks. These subgraphs must be connected components, which is a prerequisite for forming protein complexes or pathways. Our method of frequent pattern extraction involves the following three key steps: identification of node similarity, graph isomorphism test and discovery of frequent patterns.

### Identification of node similarity

Each node in a PPI network represents a unique protein. Nodes are considered similar if the proteins they represent have similar functions. We use the sequence alignment algorithm Blastclust [28] to cluster protein sequences into mutually exclusive groups. Proteins present in the same cluster are deemed functionally similar to each other and they will be assigned the same cluster ID. Blastclust is a single-linkage clustering algorithm to cluster sequences hierarchically. It begins with pair-wise alignment and places a sequence in a cluster if it matches at least one of the sequences already in the cluster. Blastclust uses the BLASTP algorithm to compute the pair-wise matches. We used stringent criteria of 90% sequence identity over 95% of the length of each sequence and divided 18,888 proteins to 14,838 clusters. The cluster ID will be tagged to each node in the network and will be used in pattern labeling process as described in the following section.

### Graph isomorphism test

The basic idea in canonical graph labeling [29] is to represent relational graph data using a sequence of symbols that can uniquely identify the graph. Kuramochi et al. [12] proposed to use concatenation of upper triangle of adjacency matrix as canonical label of graphs. For graphs with no edge weights, an adjacency matrix is a binary matrix. Every row and column corresponds to a node in the graph. The value at M[i, j] is 1 if there is an edge between node i and node j and 0 otherwise. For undirected graphs, the adjacency matrix is symmetric on its main diagonal. Therefore we can use upper right triangle of the adjacency matrix to fully represent a graph. The ordering of rows and columns will determine the content of adjacency matrix. We order the rows and columns using protein IDs the nodes are labeled with. The adjacency matrix generated in such way unambiguously represents a given graph. To create the canonical label of the graph, we first concatenate the protein IDs sorted in order. Then we concatenate the upper triangle of the adjacency matrix.

**Figure 2:**
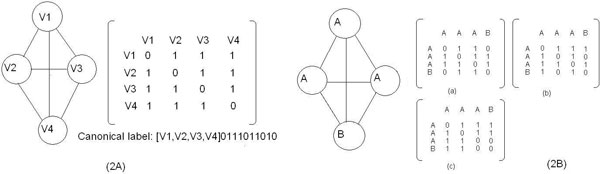
**Canonical labeling of subgraph structures**. **2A: **The columns of the adjacency matrix are arranged according to the natural order of node labels. As this is a complete graph, there are edges between every pair of distinct nodes. Therefore non-diagonal elements are all 1. And since there is no self-loop in the graph, the diagonal elements are all 0. The canonical label [V1, V2, V3, V4]0111011010 is formed of two parts. The first part [V1, V2, V3, V4] is the concatenation of node labels, delimited by comma. The second part 0111011010 is the concatenation of upper triangle of adjacency matrix. Two parts are separated by square bracket. **2B: **Three of the nodes are having same cluster ID, which results in three possible adjacency matrices to be constructed.

Figure 2A illustrates how canonical label is created for a four-node graph. If we can apply similar idea to define canonical labels of graph patterns, then graphs with same pattern labels are isomorphic to each other. Using the method described above, we can replace protein IDs with cluster IDs and generate a new series of symbols. However when there are multiple nodes bearing same cluster IDs in a graph, we cannot make a proper ordering of the nodes because different ordering of the nodes will result in different code [12]; thus making them ineffective for isomorphism test as illustrated in Figure 2B. In this Figure, three of the nodes are having the same cluster ID, 'A', which results in three possible adjacency matrices to be constructed. Correspondingly three different pattern labels can be formed. One way to obtain isomorphism-invariant codes is to try every permutation of the nodes and find lexicographically the largest or smallest code. In the above case, the pattern label constructed from matrix (c) is [A, A, A, B]0111011000, which is lexicographically the largest. But doing so will result in O(|V|!) worst case running time. To overcome this problem, here we present an algorithm that generates unique pattern labels in polynomial time.

PageRank algorithm [30] is used by Google Internet search engine to measure relative importance of web pages. The algorithm calculates a numeric value for each node to indicate its ranking in the overall network. Based on the ranking information, Google can determine which web pages are more important or more relevant and tune their search results accordingly. A similar idea can be applied to compute structural equivalence. In PageRank, all graph nodes are considered of the same type. So the ranking information solely reflects the positions of nodes in the graph. In our case, we want to first differentiate graph nodes based on their cluster ID; then differentiate the nodes based on their equivalence class (see below). To achieve this purpose, we assign weights to nodes based on their cluster ID. We associate a unique integer value with each cluster. The same integer value will be assigned to all nodes in the cluster as the weight of the node. The magnitude of the weight is not an indication of the functional importance of the cluster. It is solely used to differentiate the clusters.

**Figure 3:**
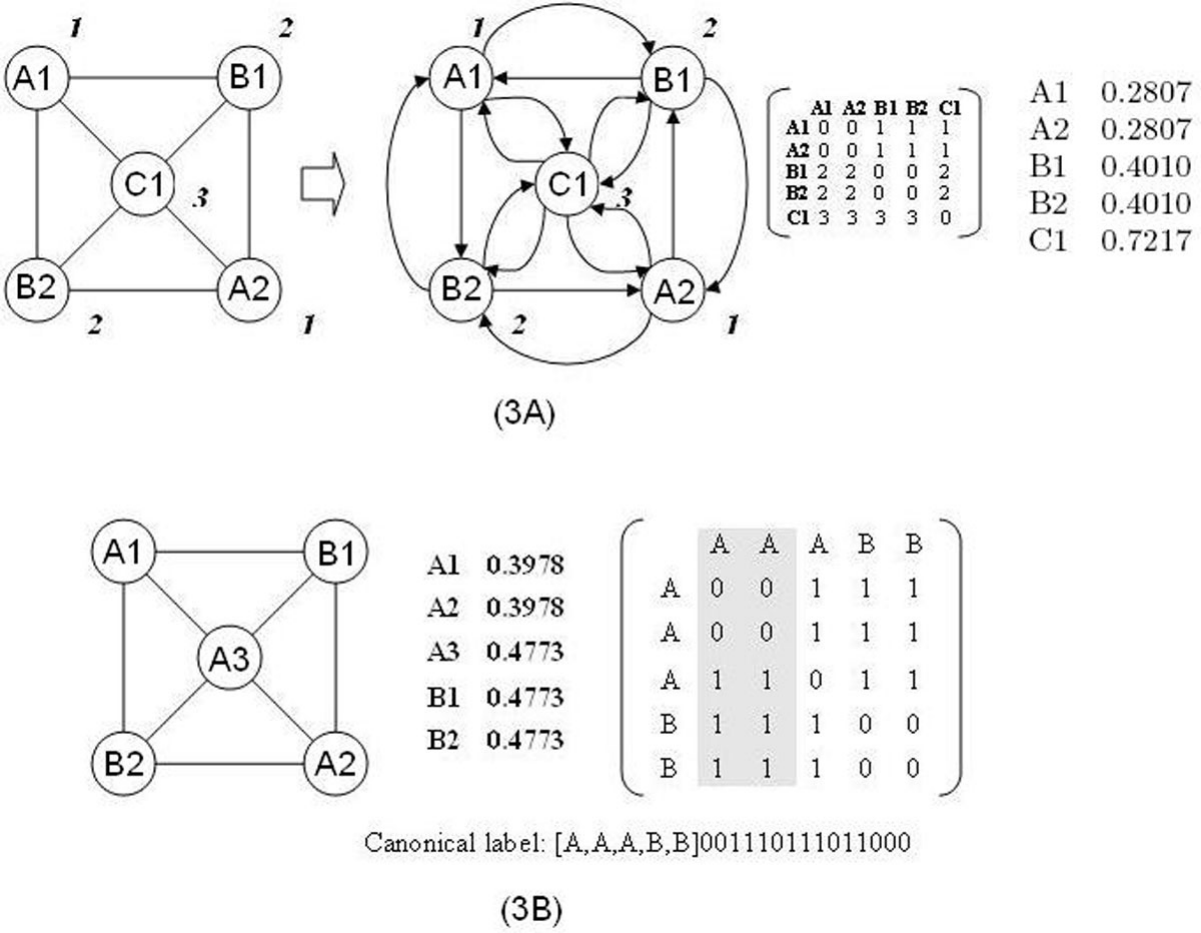
**Computing the weighted adjacency matrix**.

In Figure 3A, all nodes from cluster A are assigned weight 1; all nodes from cluster B are assigned weight 2, etc. In a weighted graph, nodes at either end of an edge are not equal because they may be assigned different weights. Therefore we replace undirected edges with two edges going to opposite directions. Then we compute the adjacency matrix, denoted as W for the weighted graph.

$$W_{\left[i,j\right]}=\left\{\begin{array}{cc} \text{weight of node i,} & \text{if node j connected to node i}\\ 0, & \text{if not} \end{array}\right)$$

From adjacency matrix, we can compute hyperlink matrix, denoted as H.

$$H_{\left[i,j\right]}=\frac{W_{\left[i,j\right]}}{\sum_{i=1}^{k}W_{\left[i,j\right]}},k\;\text{is number of nodes in graph}$$

The hyperlink matrix generated from the above example is

$$\begin{array}{c} W=\left[\begin{array}{ccccc} 0 & 0 & 1 & 1 & 1\\ 0 & 0 & 1 & 1 & 1\\ 2 & 2 & 0 & 0 & 2\\ 2 & 2 & 0 & 0 & 2\\ 3 & 3 & 3 & 3 & 0 \end{array}\right]\Rightarrow \\ H=\left[\begin{array}{ccccc} 0 & 0 & 1/5 & 1/5 & 1/6\\ 0 & 0 & 1/5 & 1/5 & 1/6\\ 2/7 & 2/7 & 0 & 0 & 1/3\\ 2/7 & 2/7 & 0 & 0 & 1/3\\ 3/7 & 3/7 & 3/5 & 3/5 & 0 \end{array}\right] \end{array}$$

Hyperlink matrix is a stochastic matrix. Every column of H sums to 1. The entry H[i, j] indicates the probability of moving from node j to node i. It can also be understood as the ratio of contribution node j makes to node i among all nodes j connected to. Let v be the vector storing relative importance of nodes. v[i] denotes the relative importance of node i. A node's relative importance is determined by the contribution all other nodes have made to it. So we need to solve the equation Hv = v. This is actually to find the Eigen vector corresponding to eigenvalue 1 of matrix H. Eigenvalue computation can be performed in polynomial time.

It shows that A1 and A2 are of the same relative importance. They will be included in the same equivalence class. B1 and B2 will also be included in the same equivalence class. Then we sort nodes based on cluster ID at first level and equivalence class at second level. In matrix M when we shuffle nodes in the same equivalence class, the matrix content will not be changed; the canonical label remains the same. Therefore permutations are not needed to generate a unique pattern label.

In Figure 3B, node A1, A2 and A3 are from the same cluster. But A3 falls into a different equivalence class from A1 and A2 because their relative importance values (the middle column) are different. When we sort the nodes, the relative positions between equivalence classes are fixed. The order is based on the relative importance value. The relative position within equivalence classes can be changed without impacting the content of matrix.

Using the algorithm described above we can generate pattern labels for graphs. Generally it takes O(n^3^) time to compute eigenvalue decomposition. Constructing adjacency matrix and hyperlink matrix each takes O(n^2^) time. Sorting of nodes takes O(n lg n) time. Thus the algorithm to compute pattern labels runs in polynomial time.

### Discovery of frequent patterns

Finding frequent subgraphs is an NP-hard problem. When the size of the subgraph is a variant, finding frequent subgraphs takes exponential run-time. Therefore, to solve frequent subgraphs problem we need to effectively reduce the search space as subgraph size increases. To accomplish this, we take the bottom up approach to find small subgraphs first and proceed to larger subgraphs. We start with frequent subgraphs of 1 node. We look for clusters with size no less than the given threshold in each network. This can be done through a simple counting of nodes within each cluster in each network. Among the selected clusters, we look for those present in all networks. Nodes belonging to these clusters are kept; the rest are removed from the networks. Edges incident to removed nodes are also removed from the networks. On the remaining part of the networks we will discover patterns of next size level.

**Figure 4:**
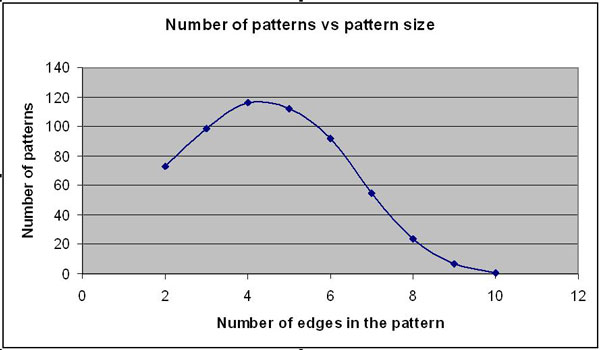
**Graph showing the number of identified patterns versus pattern size**.

Frequency downward closure is an important property that most of the frequent-subgraph-finding algorithms are based on. It is essential for the computational tractability of most frequent subgraph discovery algorithms [3]. Frequency downward closure property states that the frequency of subgraphs decreases monotonically as a function of its size. Our algorithm also looks for non-overlapping subgraphs when counting the subgraph frequency. Counting edge-disjoint embeddings of subgraph patterns can be transformed to Maximum Independent Set (MIS) problem. Pattern labels are formulated using the canonical labeling algorithm described in the previous section. Frequencies of patterns are first computed by counting the occurrence of pattern labels. Then MIS algorithm will be used to further filter overlapping patterns. Finally, we check if the selected patterns exist in all the nine cancer networks. Unqualified subgraphs will be removed from the networks. Qualified patterns will be kept for next round of pattern finding. Using these procedural steps iteratively, we have identified a number of frequent and common subgraphs at each edge-level covering from 2-10 edge subgraphs (Figure 4). A complete list of the patterns at each edge-group can be accessed from the additional files 1, 2, 3, 4, 5, 6, 7, 8, 9.

Figure 4 summarize the number of common and frequent patterns at each edge size. From 2-edge to 4-edge, the number of patterns increases as pattern size increases. In these cases, the number of patterns appears to be influenced by the possible combinations of edges, which is an increasing function of number of edges. From 4-edge on, as the number of edges increases, there is a decline in the number of patterns. This is because it's harder for large size patterns to be both frequent and common. As shown in Figure 4, the 10-edge is the maximum size of common and frequent pattern that could be found on our datasets. Beyond this point the number of patterns will become zero as the pattern size increases beyond 10 edges.

**Figure 5:**
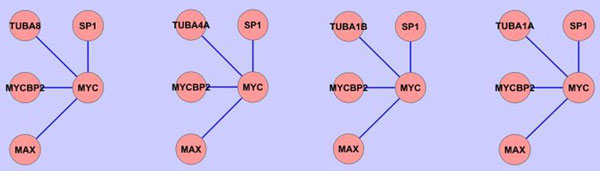
**Multiple subgraphs of the MYC pattern that vary by nodes of the same cluster at an equivalent position**. The 4 subgraphs have similar nodes (TUBA4A, TUBA8, TUBA1B and TUBA1A) at corresponding positions. Therefore they belong to the same pattern.

Each of the patterns listed in Figure 4 shows the same topology but corresponds to multiple subgraphs, where the subgraphs can vary with one another by having different nodes from the same cluster at equivalent positions. This is illustrated in Figure 5, generated in Cytoscape [31], for a 4-edge pattern involving MYC as the central node with the alpha and beta tubulins and their homologs varying in the same position. Similarly, the 10-edge pattern corresponds to 16 distinct subgraphs in bladder cancer. Note that all the common patterns exist in all the nine cancer networks, but the number of subgraphs in each pattern varies among them due to the cancer tissue-specific expression of the equivalent genes that belong to the same cluster. Patterns of smaller sizes exhibit more variations because more subgraphs are available.

### Performance validation

We compared our method with FSG, which is a frequent subgraph-mining algorithm [12], on analyzing the 9 cancer PPI networks. Given a set of network transactions, FSG looks for subgraph patterns that exist in at least σ percent of the networks, where σ is the support threshold. We ran both programs on our 24-core 2.93 Ghz Intel Xeon server. We set FSG σ to 100, which is equivalent to our method of finding common patterns in all given networks. FSG doesn't have the option of setting the subgraph support within each network and its default value is 1. At 2-edge and 3-edge levels, FSG ran faster than our method using less than one second and 1 second, respectively; while our method used 6 and 20 seconds, respectively. At 4-edge level, FSG spent similar amount of time as our method, which is around 30 seconds. But FSG was not able to continue the task at 5-and-higher-edge levels and ran out of memory. The running time and resource requirements increased exponentially as the subgraph size increased. Our method, on the other hand, showed a much slower rate of increase in time complexity. When support within network is set to 2, our program took 800 seconds to find 5-edge patterns. The running time reached the maximum for the 9-edge patterns and then finally reduced to 600 seconds at the 10-edge group.

The subgraph patterns identified by us are frequent within each network and also common to all the nine cancer networks. Hence, we hypothesize that each subgraph corresponds to an important functional module in cancer. We used GO semantic similarity [32] as a metric to quantitatively verify the functional importance of the frequent common patterns, and thus the performance of our method, in detecting the functional subgraphs. Semantic similarity provides a quantitative measure of how similar a pair of proteins is, based on the annotations (GO terms) in a given GO concept category. The idea is that the interacting proteins are more likely associated with similar cellular processes and/or involved in similar function. Hence, this similarity measure is higher for functionally related proteins, and vice versa. This concept has been very effective in interpreting the functional similarities of genes/proteins based on gene annotation information from heterogeneous data sources [33,34].

**Figure 6:**
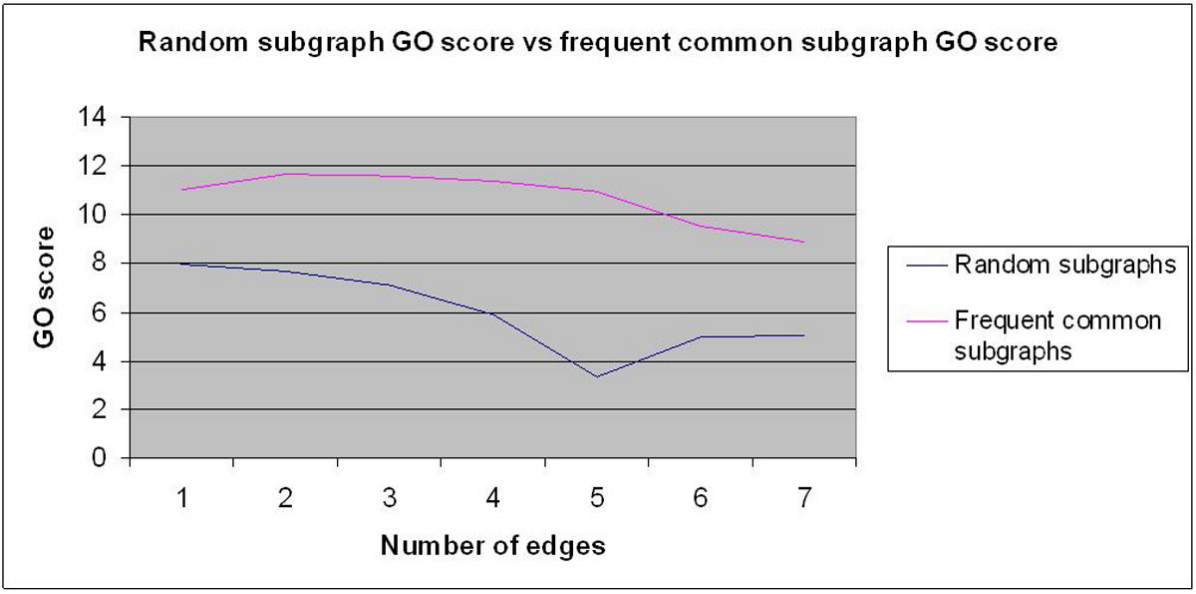
**Validation of the prediction performance using GO semantic similarity scores**. The purple line represents average GO scores of cancer subgraphs and the blue line represents those of randomly generated subgraphs, at each edge-group level.

To test this hypothesis, we compared sets of randomly generated subgraphs (SG_Rand_) against the sets identified by our algorithm (SG_Cancer_). We generated random sets of 1000 subgraphs for each edge-group of size n (n = 4-10) from the human PPI network. In other words, both sets of SG_Rand and _SG_Cancer _subgraphs are derived from the same parent interactome, but they differ in the node and edge topologies they contain. We computed the average semantic similarity scores of SG_Rand _and SG_Cancer _subgraphs for each edge-group. The results of the comparison are shown in Figure 6. As expected, the similarity scores of SG_Cancer _subgraphs are substantially higher than those of the SG_Rand _subgraphs at all edge-group levels tested. This result validates that the SG_Cancer _subgraphs identified by our algorithm are functionally coherent modules. Still, the question remains as to what kind of a role do they play in cancer. To address this, we have further studied a select set of subgraphs from different edge-groups to understand their role in different cancers.

### Role of subgraph patterns in cancer

**Figure 7:**
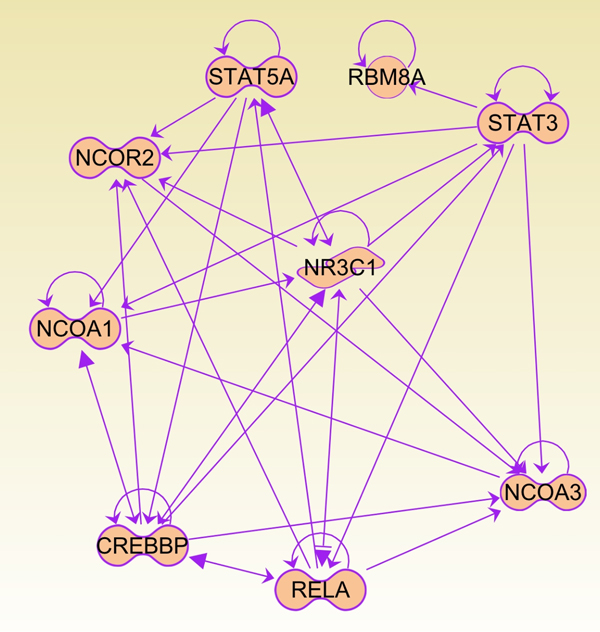
**Ingenuity pathway analysis of the 10-edge pattern subgraph showing cancer-associated interactions among its nodes**. The edges represent both physical (direct) and regulatory (indirect) relationships.

The 10-edge subgraph primarily consists of the glucocorticoid receptor (NR3C1), three of its coactivators (CREBBP, NCOA1, and NCOA3) and one co-repressor (NCOR2). In addition, there are three transcriptional regulators (STAT3, STAT5A and RELA) and an RNA binding motif protein (RBM8A). All the known direct and indirect interactions among these proteins are shown in Figure 7, which is generated by the Ingenuity Pathway Analysis tool (IPA) using only the "cancer disease" filter. All nine nodes identified in our 10-edge pattern subgraph are associated with the cancer disease with glucocorticoid receptor (GR) as the central molecule. GR plays a prominent role in apoptosis through genomic [35] and non-genomic [36] mechanisms. Due to this action of GR, glucocorticoids are commonly used to treat patients suffering from a wide range of cancers [35]. All the three coactivators of GR exhibit histone acetyl transferase activity (HAT), and genetic alterations in HATs have been linked to various forms of cancer [37]. For example, NCOA1 (SRC-1) and NCOA3 (SRC-3) are members of the p160/steroid receptor coactivator (SRC) family that are the most studied of all transcriptional coactivators [38]. SRC genes are subject to amplification and overexpression in some breast and prostate cancers [39]. The role of the third coactivator, CREBBP (CBP), merits special mention: its role in tumor suppression has been well-documented [40], and in a recent study, sequence or deletion mutations of CREBBP was found to be highly associated with relapsed acute lymphoblastic leukemia, a leading cause of death due to disease in young people [41]. CREBBP also regulates the tumor suppressor p53 in two ways: in the nucleus, acetylation of p53 by the HAT domain activates p53 [42] through formation of a binary complex [43]. In the cytosol, CREBPP promotes polyubiquitination and destabilization of p53 [44]. The RNA-binding motif containing gene, RBM8A is also known to interact with OVCA1, which is a tumor suppressor gene [45]. In summary, the functional module highlighted in this study directly impacts the activity of the Glucocorticoid Receptor, and its dysregulation, probably through the effect on the GR mediated apoptosis pathway, is a common motif found in the nine cancers included in this study. This functional module also impacts the p53 mediated tumor suppressor pathway through the regulation of p53 activity by CREBBP.

We also looked at some of the smaller subgraphs containing 2-8 edges and found a number of network patterns associated with cytoskeletal functions. One of the 8-edge patterns is related to a functional unit consisting of actin (α, β and γ isoforms) and six actin associated genes, ACTR1A, CCT5, GSN, SPTAN1, TPM1, DYNLL1 and their homologs, that are differentially expressed across nine cancer types. CCT5 is a molecular chaperone, and is part of the TCP1 ring complex, known to fold various proteins including actin and tubulin. We find that CCT5 is uniformly up-regulated across datasets. We hypothesize that CCT5 may play an important role in ensuring the correct folding of cytoskeletal proteins that are produced during cell proliferation in cancer. It is well known that the actin cytoskeleton is substantially modified in transformed cells, and this occurs in concert with changes in a host of actin filament-associated regulatory proteins [46]. These changes are thought be integrally involved in the abnormal growth properties of tumor cells, their ability to adhere to tissue, and their increased ability to metastasize [47].

In the 5-edge group of patterns, we have identified a functional module centered on the well-known oncogene MYC, and Myc binding proteins, Max, Mycbp2 (PAM), and SP1, that are differentially regulated in nine cancers. Interestingly, this functional pattern also includes α and β tubulins and their homologs in various subgraphs as shown in Figure 5. The MYC proto-oncogene family has been the subject of intense scrutiny due to the involvement of deregulated MYC genes in a wide range of cancers [48]. Myc is a short-lived protein that promotes proliferation by regulating the expression of specific target genes. Myc requires the constitutively expressed family member Max to function. Myc and Max form heterodimers via basic helix-loop-helix leucine zipper domains and bind to E-box regulatory elements in target genes. Myc overexpression up-regulates genes directed towards cell growth: ribosome biogenesis, protein synthesis, and metabolism [49], and Myc has also been shown to repress genes that attenuate cell cycle progression [50]. High-throughput sequencing of ChIP DNA (ChIP-seq) has been used to locate 3465 DNA regions bound by Myc, 20% of which were up or down-regulated as a consequence of c-Myc expression [51]. Oncogenic activation is known to occur from constitutive and overexpression of the c-Myc protein. For example, in Burkitt's lymphoma, a translocation of MYC, t(8,14) to a location that falls within the regulation of the strong promoter of immunoglobin genes increases the amount of expression of the MYC gene.

## Conclusion

In this paper, we present a novel algorithm for mining frequent and common patterns across multiple cancer PPI networks. The comprehensive PPI datasets used in this study exhibit power-law distribution across all cancer networks. By using effective canonical labeling and adopting weighted adjacency matrices, we are able to perform graph isomorphism test in polynomial running time. The search starts from small patterns of 1 node, proceeds by incrementing the subgraph size 1 edge at a time, and stops when no frequent patterns are discovered for a certain edge level. As the size increments, the infrequent edges in the original networks are removed, thus reducing the search space for the next round of searching. We applied the algorithm on nine cancer PPI networks and identified frequent and common patterns of different sizes up to 10 edges. To validate the performance of our method, we compared these patterns against the randomly generated patterns at each edge-group, using GO semantic similarity measure. Patterns identified in this study exhibited significantly higher scores compared to the random ones at all edge-group levels indicating that these patterns are functionally cohesive modules. Further investigations on the specific role of each module in cancer revealed their intricate association with various cancer-associated processes such as transcriptional regulation, cell growth, cell proliferation, etc. Ingenuity pathway analysis of a 10-edge module demonstrated that the cancer-associated functions are tightly dependent among the nodes of the subgraph as evidenced by both direct and interactions. Based on these results, we believe that the methodology developed in this study is capable of identifying common and frequent subgraphs from large and multiple interaction networks. While we used cancer PPI networks in our study, this is a generic methodology and hence can be applied to mine subgraphs from many other networks.

## Methods

### Human protein interactome dataset

We created a comprehensive, non-redundant dataset of experimentally-derived interacting proteins by combining multiple datasets (downloaded in the PSI MI 2.5 format) from five major protein interaction databases that include DIP (Database of Interacting Proteins) [26], IntAct [23], BIND (Biomolecular Interaction Network Database) [27], HPRD (Human Protein Reference Database) [25] and MINT (Molecular Interaction database) [24]. These datasets are fairly overlapping both within and across databases, and protein sequences in these databases are originally indexed with different source identifiers from UniProt, DIP, GenBank, etc. We have collected only those proteins belonging to the human species. To remove redundancy, we first created datasets of unique sequences (based on full-length protein sequence string comparison) within each database and then merged them to create a non-redundant dataset of interacting protein sequences, each indexed with our internal identifier. Finally, we obtained 19,710 unique protein sequences representing 95,931 unique PPIs.

### Calculation of GO semantic similarity

The semantic similarity of GO terms between two interacting proteins was calculated for all possible pairs of proteins in the human PPI network. The GO terms associated with each protein were obtained from the GO database. The GO annotation (GOA) for a protein can be based on three concepts i.e., biological process (P), molecular function (F) and cellular component (C). The best semantic similarity measure between the GO terms of the two proteins, under each GO concept, was determined for all pairs of proteins using the method proposed by Brown and Jurisica [33].

Semantic similarity is the probability of minimum subsumer, *P_ms _*that is determined separately for each GO concept using the following derivation. Let *g_1 _*and *g_2 _*represent the set of GO terms from proteins *i *and *j*, respectively; let *S(g_1_, g_2_) *represent the set of shared parental GO terms of *g_1 _*and *g_2_*, and let Gc represent GO concept P, F or C. Then, *P_ms _*is calculated as the minimum frequency of occurrence of the set of shared GO terms over each concept:

$$P_{ms}\left(g_{1},g_{2}\right)=\underset{S\left(g_{1},g_{2}\right)|Gc}{\text{min}}\left\{p\left(g_{i}\right)\right\}$$

A similarity measure based on this probability is then calculated as the negative log probability of minimum subsumer, using the following equation.

$$Sim\left(g_{1},g_{2}\right)=-\text{ln}\left(P_{ms}\left(g_{1},g_{2}\right)\right)$$

In brief, the similarity score between two GO terms is higher if they share a common parent with a more specific GO term (less frequent), and vice versa. The total similarity score is the sum of the best similarity scores from each concept.

### Graph theory preliminaries

Definition 1 (Labeled graph) A labeled graph is a triple G = (V, E, μ), where

• V is the node set

• E is the edge set, E ⊆ V × V

• μ:V → L_V _is a function assigning labels to nodes

In PPI networks, nodes are labeled with protein IDs. Since each protein appears at most once in a PPI network, no two nodes share same labels. Formally: ∀ v_i_, v_j _∈ V, v_i _≠ v_j _→ μ(v_i_) ≠ μ(v_j_).

Definition 2 (Undirected graph, connected graph) A graph G = (V, E, μ) is an undirected graph if and only if

∀v_i_, v_j _∈ V: (v_i_; v_j_) ∈ E ↔ (v_j _; v_i_) ∈ E. In an undirected graph G, two nodes v_i _and v_j _are connected if G contains a path from v_i _to v_j_. A graph is said to be connected if every pair of nodes in the graph are connected.

Definition 3 (Subgraph) Graph G' = (V', E', μ') is a subgraph of graph G = (V, E, μ) if V' ⊆ V and E' ⊆ (V' × V') ∩ E) and μ' = μ.

Definition 4 (Graph isomorphism) Given two labeled graphs G = (V, E, μ) and G' = (V', E', μ'). Graph isomorphism is a bijective function f: V → V' such that ∀v_i_, v_j _∊ V, (v_i_, v_j_) ∊ E ↔ (f(v_i_), f(v_j_)) ∊ E'.

Definition 5 (Frequent subgraph) Given a graph G = (V, E, μ), support(g) is the number of isomorphic embeddings of subgraph g. A subgraph is frequent if its support is no less than a given minimum support threshold.

### Algorithms

**Algorithm 1 **frequentCommonDiscover(G,σ)

1: for Every G_i _in G do

*2:   C_i _← Find node clusters with size no less than *σ

3: end for

4: F^0 ^← Find node clusters that are present in all C_0 _~ C_k_

   //k is number of graphs in G

5: for Every G_i _in G do

6:   Remove nodes not present in clusters in F^0^

7: end for

8: for Every G_i _in G do

9:   Label edges with concatenation of sorted label of nodes at both ends

10:   Label edge groups with concatenation of sorted cluster ID of nodes at both ends

*11:   L_i _← Find edge groups with size no less than *σ

12: end for

13: F^1 ^← Find edge groups that are present in all L_0 _~ L_k_

14: for Every G_i _in G do

15:   Remove edges not present in groups in F^1^

16: end for

17: t ← 2

18: while F^t-1 ^is not empty do

19:   for Every G_i _in G do

20:      E ← Enumerate t number of edges

21:      for Every E_j _in E do

22:         if connected then

23:            Assign canonical labels to subgraphs using subgraphLabel(E_j_)

24:            Assign pattern labels to subgraphs using patternLabel(E_j_)

25:         end if

26:      end for

27:      Compute embeddings of patterns using MIS()

*28:      Pi ← Find subgraph patterns with embeddings no less than *σ

29:   end for

30:   F^t ^← Find subgraphs patterns that are present in all P_0 _~ P_k_

31:   for Every G_i _in G do

32:      Remove subgraphs not present in patterns in F^t^

33:   end for

34:   t ← t + 1

35: end while

**Algorithm 2 **patternLabel(E)

1: Extract node set N from E

2: Assign weights to nodes based on their cluster ID

3: Construct weighted adjacency matrix

4: Construct hyperlink matrix

5: Compute eigenvalue decomposition of hyperlink matrix

6: Sort nodes by cluster ID first

7: Within cluster, sort nodes by corresponding values in eigen vector

8: Construct binary adjacency matrix, with nodes in order

9: Concatenate node list and upper diagonal of binary adjacency matrix

10: Return the sequence of symbols

## Author information

RS is a graduate student in CG's lab with training in computer science and this work is part of her dissertation research. NCWG is an Associate professor with training in biochemistry and molecular biology. CG (Associate professor) has an interdisciplinary background in molecular and computational biology. He has published a number of computational methods with a variety of applications in biomedical research, since 2001.

## Competing interests

The authors declare that they have no competing interests.

## Authors' contributions

RS carried out this work, developed the method, analyzed the results and drafted the manuscript. NG assisted in the functional analysis of the identified subgraphs and in manuscript preparation. CG conceived of the study, provided overall conceptual framework for this paper, analyzed the results and wrote part of the manuscript. All authors have read and approved the final manuscript.

## Supplementary Material

Additional file 1 **List of 2-node subgraphs**. Click here for file

Additional file 2 **List of 3-node subgraphs**. Click here for file

Additional file 3 **List of 4-node subgraphs**. Click here for file

Additional file 4 **List of 5-node subgraphs**. Click here for file

Additional file 5 **List of 6-node subgraphs**. Click here for file

Additional file 6 **List of 7-node subgraphs**. Click here for file

Additional file 7 **List of 8-node subgraphs**. Click here for file

Additional file 8 **List of 9-node subgraphs**. Click here for file

Additional file 9 **List of 10-node subgraphs**. Click here for file
